# Development of a multiplex real-time PCR assay for detection of *Mycoplasma pneumoniae*, *Chlamydiapneumoniae* and mutations associated with macrolide resistance in *Mycoplasma pneumoniae* from respiratory clinical specimens

**DOI:** 10.1186/s40064-015-1457-x

**Published:** 2015-11-10

**Authors:** Maaret Nummi, Laura Mannonen, Mirja Puolakkainen

**Affiliations:** Virology and Immunology, University of Helsinki and Helsinki University Hospital, P.O. Box 400, 00029 HUS Helsinki, Finland

**Keywords:** Multiplex real-time PCR, *Mycoplasma pneumoniae*, Macrolide resistance, *Chlamydia pneumoniae*

## Abstract

The aim of this study was to improve detection of *Mycoplasma pneumoniae* and *Chlamydia pneumoniae* in clinical specimens by developing a multiplex real-time PCR assay that includes identification of macrolide-resistant *M. pneumoniae*. Novel assays targeting a *M. pneumoniae* conserved hypothetical protein gene, *M. pneumoniae* 23S rRNA gene mutations associated with macrolide resistance and human β-globin gene (an endogenous internal control) were designed and combined with a previously published *C. pneumoniae* PCR targeting ompA gene. The resulting quadraplex PCR was validated with a panel of clinical specimens supplemented with external quality assessment specimens, simulated specimens and various bacterial and viral strains. The obtained results were compared to those obtained by reference PCRs or confirmed by sequencing (typing of macrolide resistance). The novel multiplex PCR assay was in 100 % agreement with reference PCRs. Four *M. pneumoniae* strains with macrolide resistance-associated mutations were identified among 42 strains, which comprises 9.5 % of the study material. Amplification of an internal control excluded sample-derived inhibition possibly leading to false-negative reporting. In conclusion, we have developed a resources conserving multiplex real-time PCR assay for simultaneous detection of *M. pneumoniae*, *C. pneumoniae* and the most common mutations leading to macrolide resistance in *M. pneumoniae*. The assay is a widely useful tool for detection of these respiratory pathogens and will also shed light on the occurrence of macrolide resistance in *M. pneumoniae.*

**Electronic supplementary material:**

The online version of this article (doi:10.1186/s40064-015-1457-x) contains supplementary material, which is available to authorized users.

## Background

*Mycoplasma pneumoniae* and *Chlamydia pneumoniae* are common respiratory pathogens causing approximately 10–30 % and 5–10 %, respectively, of community acquired pneumonias (Atkinson et al. 2008; Kuo et al. 1995). *M. pneumoniae* infections can also present with extrapulmonary symptoms, including mucocutaneous and central nervous system manifestations (Atkinson et al. 2008), and the clinical spectrum of *C. pneumoniae* infection includes reactive arthritis, worsening of asthma and cardiovascular disease (Orrskog et al. 2013).

*Mycoplasma pneumoniae* infection can occur in all age groups, but the severity of the disease tends to increase with the age of the patient. For immunocompromised patients, *M. pneumoniae* infection can lead to a severe pneumonia (Waites and Talkington 2004). *C. pneumoniae* IgG antibody prevalence bounces between 5 and 15 years of age and then increases steadily suggesting that most primary infections take place in school-age children and repeated infections occur later in life. Antimicrobial therapy is available for *M. pneumoniae* and *C. pneumoniae* infection. In case antimicrobial treatment is indicated, specific and rapid laboratory diagnosis is important as the diagnosis cannot be based on clinical symptoms only. *M. pneumoniae* pneumonias are often treated with macrolides but also tetracyclines and fluoroquinolones are effective (Waites and Talkington 2004). Macrolides are often used for treatment due to their safety especially for children and pregnant women. However, in the last few years macrolide-resistant *M. pneumoniae* strains have emerged, with high rates in China and Japan, and lower rates in Europe and Canada (Principi and Esposito 2013). Resistance to macrolides is caused by point mutations in the bacterial 23S rRNA gene which reduces the affinity of the antibiotic for the ribosomes (Wolff et al. 2008). The most common point mutations leading to the resistance are located in the 23S rRNA gene at positions 2063 and 2064, and include transition A to G or C (Waites and Talkington 2004; Lucier et al. 1995).

In recent years, nucleic acid-based assays targeting *M. pneumoniae* and *C. pneumoniae* have been developed (Dumke and Jacobs 2009, 2014; Winchell et al. 2008). PCR-based methods have high sensitivity, short turnaround time and the testing can be completed in a single working day. Moreover, simultaneous amplification of more than one target in the same reaction (multiplex PCR) is becoming a rapid, economical and convenient means in a diagnostic laboratory to detect multiple pathogens causing similar symptoms and their genetic properties at the same. A variety of multiplex PCRs have been developed for detection of *M. pneumoniae* and *C. pneumoniae* (Thurman et al. 2011; Welti et al. 2003; Gullsby et al. 2008; Miyashita et al. 2004; McDonough et al. 2005). However, also molecular diagnosis has limitations since the mere presence of nucleic acid can denote either acute infection, persistence of nucleic acid after infection or asymptomatic carriage.

The aim of this study was to improve detection of *M. pneumoniae* and *C. pneumoniae* in clinical specimens by developing a time-saving and inexpensive multiplex real-time PCR assay for detection of *C. pneumoniae*, *M. pneumoniae* and the most common mutations in the M*. pneumoniae* 23S rRNA gene leading to resistance for macrolide antibiotics. To our knowledge, this is the first report of a multiplex real-time PCR assay that in addition to the detection of *M. pneumoniae, C. pneumoniae* and internal control includes detection of macrolide resistance-associated mutations in *M. pneumoniae*.

## Results

### Sensitivity of co-amplification

To assess the sensitivity of co-amplification of *C. pneumoniae*, *M. pneumoniae* and human beta-globin by multiplex real-time PCR, a range of concentrations of *M. pneumoniae* M129 and *C. pneumoniae* K-6 DNA were co-amplified. In addition, low concentrations of *M. pneumoniae* and *C. pneumoniae* were amplified in presence of high concentration of human DNA. Crossing threshold (Ct) values remained unchanged for the DNA that was in a lower concentration even when a high concentration of DNA from another organism was co-amplified (data not shown).

### Internal control (IC) human beta-globin

The human β-globin was co-amplified in the multiplex reaction as IC. Neither sample matrix-derived inhibition in the PCR reaction, nor specimen processing errors potentially leading to false negative reporting were observed in this study. The Ct values in nose and/or throat swabs (n = 72) varied between 18.9 and 28.1 (mean 23.2, median 23) and for BALs (n = 13) the Ct range was 21.6–29.6 (mean 25.3, median 26).

### Analytical sensitivity

The analytical sensitivity, the limit of detection (LOD) with 95 % probability, was determined for *M. pneumoniae* and *C. pneumoniae* components of the multiplex real-time PCR using Probit analysis of the Validation Manager software (Finbiosoft, Espoo, Finland). The analytical sensitivity for *M. pneumoniae* was 2.9 copies/PCR-reaction and for *C. pneumoniae (K*-*6)* the LOD was 0.13 IFU/PCR reaction. To confirm that the *C. pneumoniae* primers and probe are able to detect different strains, DNA from nine available *C. pneumoniae* isolates were tested: The strains isolated in Finland K6, K8, K66, FIL, HELS-12, MS and Parola, CV6 from Germany (a kind gift from Prof. M Maass), and TW-183 (ATCC VR-2282) and CWL-029 (ATCC VR-1310). The multiplex PCR detected these strains.

### Analytical specificity

The specificity of the primers and probes was evaluated using BLAST analysis, and analyzing nucleic acid from various bacterial and viral strains in the multiplex real-time PCR (Additional file 1, Table S1). *In silico* and in vitro analyses revealed that the *M. pneumoniae* primers and probe designed for detection of the most common resistance-associated mutations could also amplify DNA from *Mycoplasma genitalium.*

### Diagnostic performance of the multiplex PCR

To evaluate the reliability of the developed real-time multiplex PCR for clinical testing the performance of the assay was compared to those of the reference PCR assays. A total of 42 *M. pneumoniae* positive specimens and 24 *M. pneumoniae* simulated BALs were studied with the multiplex assay. The multiplex real-time PCR detected all simulated BALs correctly and the assay was in 100 % agreement with the reference PCRs (Table 1).

**Table 1:** Comparison of the results from multiplex real-time PCR and reference PCRs for detection of (a) *M. pneumoniae* (MP) and (b) *C. pneumoniae* (CP) in clinical respiratory specimens

*M. pneumoniae* (MP)		Reference PCRs		Total
		MP pos	MP neg	
Multiplex PCR	MP pos	42	0	42
	MP neg	0	43	43
	Total	42	43	85

*C. pneumoniae* (CP)		CP pos	CP neg	Total
Multiplex PCR	CP pos	28	0	28
	CP neg	0	43	43
	Total	28	43	71

**Fig. 1:**
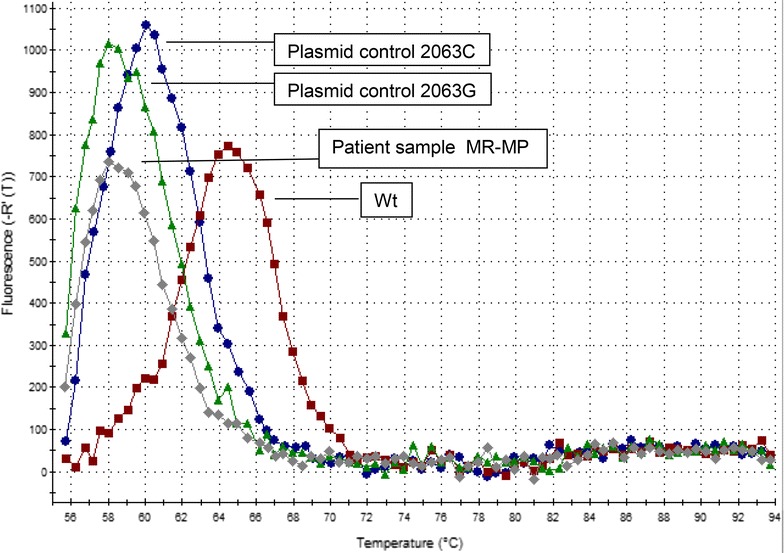
Melting curve displaying a patient sample containing DNA from *M. pneumoniae* with macrolide resistance-associated mutation (MR–MP). The melting profile of a patient sample was compared to the melting profiles of 3 controls: (1) *Plasmid control 2063C* which contains *M. pneumoniae* 23S rRNA PCR target sequence with base C at the position 2063 (DNA amplicon with base C at the position 2064 displays similar melting profile, data not shown). (2) Plasmid control 2063G which contains *M. pneumoniae* 23S rRNA target sequence with base G at the position 2063 (DNA amplicon with base G at position the 2064 displays similar melting profile, data not shown). (3) *Wt* (wild type) *M. pneumoniae* DNA

The macrolide resistance-associated mutations in *M. pneumoniae* strains were identified by dissociation curve analysis of the amplicon. The analysis distinguishes mutations in 23S rRNA sequence at positions 2063G/C or 2064G/C. The melting temperature of the wild type is 64–65 °C, for the mutation C 60–61 °C and for the mutation G 57.5–58.5 °C (Fig. 1). The dissociation curve analysis does not differentiate whether the mutation is in the base 2063 or 2064. In our study material, 9.5 % (4/42) of the *M. pneumoniae* positive specimens contained a *M. pneumoniae* strain with macrolide resistance-associated mutation. The PCR results were confirmed by sequence analysis. Two BALs and two sputum specimens contained *M. pneumoniae* with a mutation associated with macrolide resistance. Three *M. pneumoniae* strains had a point mutation at 2063 A->G and one sputum specimen contained both wild type DNA and DNA with a point mutation at 2064 A->G.

The *C. pneumoniae* component in the multiplex PCR uses same oligonucleotides as the previously validated reference PCR and hence the performance of the *C. pneumoniae* component was verified with a smaller amount of specimens. The clinical sensitivity was verified by testing 28 *C. pneumoniae* positive or simulated positive upper and lower respiratory tract specimens. In addition, a total of 43 *C. pneumoniae* negative respiratory tract specimens were assessed. The multiplex PCR assay was in 100 % agreement with the reference method (Table 1).

### External quality assessment panel

EQA panel *C. pneumoniae* and *M. pneumoniae* 2014 (QCMD, Glasgow, Scotland, UK) was analyzed with the multiplex real-time PCR assay. The multiplex PCR identified all core proficiency samples correctly. Only two educational, infrequently detected *M.pneumoniae* positive specimens were not detected by the multiplex PCR (CP.MP14-03 and CP.MP14-12) and one educational infrequently detected *C. pneumoniae* positive specimen CP.MP14-06 gave a negative result (Table 2).

**Table 2:** The results of QCMD 2014 proficiency panel for *C. pneumoniae* and *M. pneumoniae* using the developed multiplex PCR method

Sample	Matrix	Sample content	Result with the multiplex-PCR	Sample status/percentage of laboratories that reported positive result for the pathogen in question	Sample type
CP.MP14-01	BAL	*C. pneumoniae*	*C. pneumoniae*	Frequently detected/96.2 %	Core
CP.MP14-02	TM	*M. pneumoniae*	*M. pneumoniae*	Detected/73.9 %	Educational
CP.MP14-03	TM	*M. pneumoniae*	Negative	Infrequently detected/20.6 %	Educational
CP.MP14-04	TM	Negative	Negative	Negative	Core
CP.MP14-05	TM	*C. pneumoniae*	*C. pneumoniae*	Frequently detected/98.4 %	Core
CP.MP14-06	TM	*C. pneumoniae*	Negative	Infrequently detected/58 %	Educational
CP.MP14-07	BAL	Negative	Negative	Negative	Core
CP.MP14-08	BAL	*M. pneumoniae*	*M. pneumoniae*	Detected/78.4 %	Educational
CP.MP14-09	TM	*C. pneumoniae*	*C. pneumoniae*	Detected/82.5 %	Educational
CP.MP14-10	BAL	*C. pneumoniae*	*C. pneumoniae*	Frequently detected/98.4 %	Core
CP.MP14-11	TM	*M. pneumoniae*	*M. pneumoniae*	Detected/94.5 %	Core
CP.MP14-12	BAL	*M. pneumoniae*	Negative	Infrequently detected/43.7 %	Educational

*BAL* Bronchoalveolar lavage, *TM* transport medium, *Core* sample type that all participants are expected to detect correctly, *Educational* sample type that is designed to provide educational information to participants

## Discussion

*Mycoplasma pneumoniae* and *C. pneumoniae* cause atypical pneumonia and have clinically similar respiratory manifestations. Hence it is logical to detect them simultaneously. To improve laboratory diagnosis and to reduce the cost and time for detecting *M. pneumoniae* and *C. pneumoniae*, we designed and validated a multiplex real-time PCR that can also detect the most common mutations in the 23S rRNA gene of *M. pneumoniae* leading to macrolide resistance in a single reaction. Human beta-globin gene was included as an internal control to allow assessment of the specimen quality and the nucleic acid extraction as well as the amplification processes. No inhibition or specimen processing errors were identified in this study. The variation of the detected IC Ct values for swab specimens and BAL specimens follows normal distribution (mean and median are almost identical) and is therefore more likely to reflect variation in the sampling procedure.

All specimens were analysed both in singleplex and in multiplex reactions. Multiplex PCR may lack sensitivity when compared to singleplex PCR (Mannonen et al. 2012), but here the carefully optimized multiplex PCR had identical sensitivity in comparison to the singleplex PCR (data not shown). The obtained LOD 2.9 cp/reaction for *M. pneumoniae* can be considered functional and is comparable to those reported by others (Dumke and Jacobs 2009, 2014; Welti et al. 2003; Schmitt et al. 2013). For the *C. pneumoniae* component of the multiplex PCR, the LOD of 0.13 IFU/PCR reaction was obtained. The sensitivities of previously published PCR assays are challenging to compare since no standard preparations for measuring analytical sensitivity are available (Dowell et al. 2001). Until international molecular standards become available, molecular EQA panels may be the best way to estimate sensitivity of a molecular test. Here we analysed a QCMD 2014 proficiency panel for *C. pneumoniae* and *M. pneumoniae* with the developed multiplex PCR method. The multiplex PCR method identified all core samples correctly indicating acceptable level of proficiency. In addition, 3/6 educational samples were identified correctly. The remaining three educational samples which tested negative by the multiplex PCR and the reference methods, were infrequently detected by the proficiency panel participants, and only 20.6 and 43.7 % of the laboratories reported positive *M. pneumoniae* results and 58 % positive *C. pneumoniae* result for these samples indicating very low level of bacteria in these samples. Overall, the developed multiplex real-time PCR showed good specificity and sensitivity for detecting *M. pneumoniae* and *C. pneumoniae* in clinical and simulated specimens. The performance of the multiplex PCR assay was comparable to that of the reference PCRs.

Macrolides are considered the treatment of choice for *M. pneumoniae* infections. Since 2000, *M. pneumoniae* strains resistant to macrolides have emerged (Bébéar et al. 2011; Principi and Esposito 2013). Mutations in the 23S rRNA gene can confer resistance to macrolides and the most commonly occurring mutations are A2063G and A2064G. In our study, 9.5 % of *M. pneumoniae* strains were shown to have either of these mutations. The observed rate is similar to those reported in other European countries and USA (0–26 %), whereas in Japan and China, the occurrence of macrolide resistance is much higher (>90 %) (Bébéar et al. 2011; Principi and Esposito 2013). A2063G is the most commonly detected mutation worldwide (>90 % of all reported point mutations), and this was also the case in our study (three out of four cases had A2063G mutation). *In silico* and in vitro studies revealed that the primers and the probe for macrolide resistance mutations also detected *M. genitalium*. The 23S rRNA genes of *M. pneumoniae* and *M. genitalium* are closely related (96.7 % identity) and therefore the observed cross-reactivity is not surprising (Dumke et al. 2010). *M. genitalium*, however, is not frequently detected in respiratory specimens. Moreover, the presence of *M. pneumoniae* is first identified by specific oligonucleotides and the presence of mutation is evaluated only in *M. pneumoniae* positive specimens, so the risk of incorrect identification was considered minimal.

As antimicrobial therapy effective against *M. pneumoniae* and *C. pneumoniae* infections is available and recommended in lower respiratory tract infections, molecular testing can aid in patient management by providing a microbiological diagnosis and guiding antibiotic selection. Sensitive nucleic acid based detection techniques also contribute to a better understanding of the epidemiology and manifestations of these infections.

## Conclusions

We have developed a resources conserving multiplex real-time PCR assay which has been successfully implemented in a clinical diagnostic laboratory for simultaneous detection of *M. pneumoniae*, *C. pneumoniae* and the most common mutations leading to macrolide resistance in *M. pneumoniae*. The assay is a widely useful tool for detection of these respiratory pathogens and will also shed light on the occurrence of macrolide resistance in *M. pneumoniae.*

## Methods

### Design of primers and probes

Primer Express software v 3.0 (Applied Biosystems, Foster City, CA) was used to design primers and probes targeting a conserved hypothetical protein C985_0367 of *M. pneumoniae* (MP) (MP M129-B7) and human β-globin (BG) gene. The *C. pneumoniae* (CP) primers and probe have previously been described by Mannonen et al. 2004. The primers and probe for detection of macrolide resistance-associated mutations in *M. pneumoniae* (MR–MP) were designed by TIB MOLBIOL (Berlin, Germany). All primers and probes were tested for specificity by basic local alignment search tool (BLAST). The primer and probe sequences are listed in Table 3.

**Table 3:** Primer and probe sequences for amplification of *C. pneumoniae, M. pneumoniae,* macrolide resistance associated mutations in *M. pneumoniae* and human beta-globin DNA, as well as the primer sequences used for sequencing of *M. pneumoniae* 23S rRNA

Primer/probe	Sequence 5′->3′	Gene target (reference)
MP Fw	ATGTACTATCAGCAAAAGCTCAGTATGG	*M. pneumoniae* Hyppthetical protein C985_0367 (GeneBank accesion no: AGC04275.1) (this publication)
MP Rev	CCACATACCGCTTTAAGTTAGCAA	
MP probe^a^	Cy5-CTAACCAAAACAGCCCTTCAACGGCA-Iowa black RQ-Sp	
CP Fw	AAGGGCTATAAAGGCGTTGCT	*C. pneumoniae* ompA (Mannonen et al. 2004)
CP Rev	TGGTCGCAGACTTTGTTCCA	
CP probe^a^	Tx Red-TCCCCTTGCCAACAGACGCTGG-Iowa black RQ-Sp	
BG Fw	GGTTGGGATAAGGCTGGATTATT	Homo sapiens beta-globin chain (GeneBank accesion no: AY260740.1) (this publication)
BG Rev	CAGGAGCTGTGGGAGGAAGA	
BG probe^a^	JOE/ZEN-CAAGCTAGGCCCTTTTGCTAATCATGTTCA-Iowa black FQ	
MR–MP Fw	GCTATAGACTCGGTGAAATCCAGG	*M. pneumoniae* gene for 23S rRNA (GeneBank accesion no: gi|288509|emb|X68422.1|) (this publication)
MR–MP Rev	GCTACAGTAAAGCTTCACTGGGTC	
MR–MP SimpleProbe^®^	GCGCA XI ACGGGACGGAAAGAC	
CARDS TX Fw	TTTGGTAGCTGGTTACGGGAAT	CARDS toxin gene (Winchell et al. 2008)
CARDS TX Rev	GGTCGGCACGAATTTCATATAAG	
CARDS TX probe^a^	TGTACCAGAGCACCCCAGAAGGGCT	
*M. pneumoniae* Fw diagnostic PCR	AAGGACCTGCAAGGGTTCGT	16SrRNA (van Kuppeveld et al. 1992)
*M. pneumoniae* Rev diagnostic PCR	Biotin-CTCTAGCCATTACCTGCTAA	
*M. pneumoniae* probe diagnostic PCR	ACTCCTACGGGAGGCAGCAGTA-digoxigenin	
MR Seq Fw^b^	AACTATAACGGTCCTAAGGTAGCG	23srRNA (Wolff et al. 2008)
MR Seq Rev^b^	GCTCCTACCTATTCTCTACATGAT	

^a^Dually labelled hydrolysis probe

^b^Sequencing primer

### Multiplex real-time PCR design

Endogenous IC human β-globin PCR assay was optimized as a primer limited reaction to avoid competition between other amplifications. The multiplex PCR reaction consisted of 5 µl of extracted DNA, 1 × Maxima Probe qPCR Master Mix (Thermo Scientific) 0.05 µM CP-Fw, 0.2 µM CP-Rev, 0.175 µM CP-probe, 0.250 µM MP-Fw and MP-Rev primers, 0.150 µ MP-probe, 0.05 µM MR-MP-Fw, 0.400 µM MR-Mp-Rev, 0.150 MR-MP-probe, 0.05 µM BG-Fw, 0.1 µM BG-Rev, 0.05 µM BG-probe and nuclease-free water in a total reaction volume of 25 μl.

Multiplex PCRs were performed using Stratagene Mx3005p instrument (Agilent Technologies Inc, Santa Clara, CA, United States). Positive and negative controls as well as a no template control (NTC) were included in each run. Cycling conditions were 1 cycle 95 °C for 10 min followed by 45 cycles of 95 °C for 15 s and 60 °C for 60 s. After amplification a dissociation curve analysis was performed with a thermal profile: 95 °C for 60 s, 55 °C for 30 s followed by a slow rise in the temperature (0.2 °C/s) to 95 °C with continuous acquisition of fluorescence signal.

### Reference PCRs

The *M. pneumoniae* results by the new multiplex PCR were compared to those obtained by the diagnostic *M. pneumoniae* PCR test in use at the time of the study at HUSLAB (adapted from van Kuppeveld et al. 1992) or to *M. pneumoniae* PCR targeting CARDS toxin (CARDStx) gene adapted from Winchell et al. 2008. For the diagnostic PCR, 5 µl of nucleic acid extract was amplified in a 50 µl reaction volume with 0.5 µM of primers (Table 3). Amplified products were detected by microplate hybridization (Vesanen et al. 1996). PCR products were detected with digoxigenin labelled probe (Table 3), anti-digoxigenin antibody conjugated with alkaline phosphatase and LumiPhos Plus substrate (Lumigen, Souhfield, MI, USA). For the CARDStx PCR 5 µl of nucleic acid extract was amplified in 25 µl PCR reaction with 0.25 µM of primers and 0.125 µM probe (Table 3). The real-time PCR reaction was carried out in Stratagene Mx3005p instrument.

The *C. pneumoniae* results obtained by the new multiplex PCR were compared to those of the diagnostic *C. pneumoniae* PCR test in use at the time of the study at HUSLAB (Mannonen et al. 2004) (with minor modifications). The primers and probe were the same as in the new multiplex test. Five µl of nucleic acid extract was amplified in 50 µl reaction mixture with 0.3 µM forward and reverse primer and 0.225 µM probe (Table 3).

### Specimens

Respiratory tract specimens referred to HUSLAB, Department of Virology and Immunology for testing of *M. pneumoniae*, *C. pneumoniae* and/or respiratory viruses between years 1999–2014, were analyzed in this study. The specimens included 42 *M. pneumoniae* positive specimens assessed by diagnostic PCR of that time (29) (van Kuppeveld et al. 1992) or by CARDS Tx PCR (13) (Winchell et al. 2008). The specimens were nasopharyngeal swabs (24), nasopharyngeal aspirates (5), sputum samples (6), tracheal aspirates (5) and bronchoalveolar lavages (BAL; 2). Due to the limited amount of *M. pneumoniae* positive BAL specimens, 24 negative BALs were spiked with *M. pneumoniae* positive respiratory specimens in dilutions 1:10–1:1000.

The *C. pneumoniae* positive specimens included two sputum samples and three nose and/or throat swabs, 12 simulated BALs and 11 simulated respiratory tract specimens. The simulated specimens were prepared by spiking negative BALs and respiratory tract specimens with *C.pneumoniae* strain K6 yielding 1.25 inclusion forming unit (IFU)/PCR reaction or 250 IFU/PCR reaction. Altogether 43 *M. pneumoniae* and *C. pneumoniae* negative upper and lower respiratory tract specimens were assessed in this study. In addition, the QCMDs External Quality assurance panel 2014 *Chlamydophila pneumoniae* and *Mycoplasma pneumoniae* was used to evaluate the performance of the developed multiplex real-time PCR.

### DNA extraction

DNA was extracted from respiratory specimens using MagNA Pure LC Total Nucleic Acid Isolation Kit or MagNA Pure DNA/Viral NA SV 2.0 Kit (Roche Diagnostics GmbH, Mannheim, Germany). In detail, 300 µl of specimen was lysed with 300 µl of MagNA Pure Lysis/Binding Buffer (Roche Diagnostics GmbH, Mannheim, Germany). Five hundred µl (MagNA Pure LC) or 450 µl (MagNA Pure 96) of specimen lysate was extracted with the MagNA Pure LC or the MagNA Pure 96-instrument and eluted in 50 µl of the elution buffer.

Specimens tested by the reference *M. pneumoniae* and *C. pneumoniae* PCRs had been extracted by either using MagNA Pure LC, EasyMAG instrument (bioMerieux, Marcy l’Etoile, France) or phenol–chloroform extraction. Starting volumes were 1800 µl for BALs, 200 µl for nasopharyngeal aspirates and sputum specimens, and 100 µl for nasopharyngeal swabs. Nucleic acids extracted by EasyMAG were eluted in 25 µl and those extracted by phenol–chloroform protocol were eluted either in 50 µl (nasopharyngeal swabs) or 20 µl (other specimens).

### Sequencing analysis of PCR products

Nucleotide sequence analysis of *M. pneumoniae* 23S rRNA gene (GeneBank. X68422) was performed to all specimens containing mutations associated with macrolide resistance in *M. pneumoniae* according to the developed multiplex real-time PCR. The primer sequences have been previously described (Table 3). The PCR products were sequenced in the Institute of Biotechnology at the University of Helsinki.

### Analytical sensitivity

The analytical sensitivity of the multiplex real-time PCR assay was assessed by amplifying serial dilutions of *M. pneumoniae* M129 DNA control (ZeptoMetrix Corporation, Franklin, MA) and DNA from *C. pneumoniae* K-6 (quantitated at Mirja Puolakkainen’s research laboratory). Four dilutions with ten replicates of each were amplified with the multiplex real-time PCR carried out in Mx3005p instrument. The tested *M. pneumoniae* M 129 DNA dilutions were 6.210, 3.105, 1.552 and 0.776 genome copies (cp)/PCR reaction, and *C. pneumoniae* K-6 DNA dilutions were 0.250, 0.125, 0.063 and 0.032 IFU/PCR reaction.

### Analytical specificity

To assess the analytical specificity of the multiplex PCR assay various bacterial and viral samples were studied (Additional file 1, Table S1).

## Additional file

10.1186/s40064-015-1457-x Viral and bacterial samples used to evaluate the analytical specificity of the multiplex real-time PCR. An excel table containing information on the material used to assess the analytical specificity of the multiplex PCR.
